# Dengue Contingency Planning: From Research to Policy and Practice

**DOI:** 10.1371/journal.pntd.0004916

**Published:** 2016-09-21

**Authors:** Silvia Runge-Ranzinger, Axel Kroeger, Piero Olliaro, Philip J. McCall, Gustavo Sánchez Tejeda, Linda S. Lloyd, Lokman Hakim, Leigh R. Bowman, Olaf Horstick, Giovanini Coelho

**Affiliations:** 1 Institute of Public Health, University of Heidelberg, Heidelberg, Germany; 2 Special Programme for Research and Training WHO-TDR, Geneva, Switzerland; 3 Liverpool School of Tropical Medicine, Liverpool, United Kingdom; 4 Ministry of Health, Mexico City, Mexico; 5 Public Health Consultant, San Diego, California, United States of America; 6 Ministry of Health, Kuala Lumpur, Malaysia; 7 Ministry of Health, Brasilia, Brazil; Duke-NUS GMS, SINGAPORE

## Abstract

**Background:**

Dengue is an increasingly incident disease across many parts of the world. In response, an evidence-based handbook to translate research into policy and practice was developed. This handbook facilitates contingency planning as well as the development and use of early warning and response systems for dengue fever epidemics, by identifying decision-making processes that contribute to the success or failure of dengue surveillance, as well as triggers that initiate effective responses to incipient outbreaks.

**Methodology/Principal findings:**

Available evidence was evaluated using a step-wise process that included systematic literature reviews, policymaker and stakeholder interviews, a study to assess dengue contingency planning and outbreak management in 10 countries, and a retrospective logistic regression analysis to identify alarm signals for an outbreak warning system using datasets from five dengue endemic countries. Best practices for managing a dengue outbreak are provided for key elements of a dengue contingency plan including timely contingency planning, the importance of a detailed, context-specific dengue contingency plan that clearly distinguishes between routine and outbreak interventions, surveillance systems for outbreak preparedness, outbreak definitions, alert algorithms, managerial capacity, vector control capacity, and clinical management of large caseloads. Additionally, a computer-assisted early warning system, which enables countries to identify and respond to context-specific variables that predict forthcoming dengue outbreaks, has been developed.

**Conclusions/Significance:**

Most countries do not have comprehensive, detailed contingency plans for dengue outbreaks. Countries tend to rely on intensified vector control as their outbreak response, with minimal focus on integrated management of clinical care, epidemiological, laboratory and vector surveillance, and risk communication. The *Technical Handbook for Surveillance*, *Dengue Outbreak Prediction/ Detection and Outbreak Response* seeks to provide countries with evidence-based best practices to justify the declaration of an outbreak and the mobilization of the resources required to implement an effective dengue contingency plan.

## Author Summary

An evidence-based handbook was generated to facilitate deployment of dengue surveillance and response systems for timely and effective management of outbreaks, and to identify the factors required for success. Evidence was evaluated using literature reviews, policymaker and stakeholder interviews, assessment of dengue contingency planning and outbreak management in ten endemic countries, and a statistical analysis to identify outbreak early warning signs in five countries. Best practices for managing dengue outbreaks included timely and context-specific dengue contingency plans that distinguished between routine practices and outbreak interventions, surveillance systems, outbreak definitions, alert algorithms, and managerial, clinical and vector control capacity. A computer-assisted early warning system was developed to enable each locality to develop its own context-specific scheme. Today, most countries do not have comprehensive, detailed contingency plans for dengue outbreaks, responding simply by intensifying vector control, with minimal focus on integrated management of clinical care, epidemiological, laboratory and vector surveillance, and risk communication. To rectify this, our handbook provides countries with evidence-based best practices to justify the declaration of an outbreak and for the mobilization and management of appropriate resources required to implement a dengue contingency plan.

## Introduction

Responding to the rapidly increasing public health importance of dengue, the 2002 World Health Assembly Resolution WHA55.17 urged greater commitment to dengue among Member States and throughout the World Health Organisation (WHO). One response of particular significance was the Revision of the International Health Regulations (WHA58.3) in 2005, where dengue was included as an example of a disease that would constitute a public health emergency of international concern. It was against this background that the World Health Organization’s Special Programme for Research and Training in Tropical Diseases (WHO/TDR) initiated a Dengue Scientific Working Group (SWG) of 60 experts from 20 countries, which met in October 2006 to review existing knowledge on dengue and establish priorities for future dengue research [1]. The research priorities identified were organized into four major research streams and those for dengue surveillance and outbreak response included the following primary recommendations:

➢ Development and utilization of early warning and response systems;
➢ Identification of triggers that initiate effective response to incipient epidemics;
➢ Decision-making processes that result in a declaration of a state of emergency;
➢ Analysis of the factors that contribute to the success or failure of national programs in the context of dengue surveillance and outbreak management.

At the same time, a discussion began that was centred on the need for an evidence base to better inform policy recommendations. The WHO Dengue Guidelines for Diagnosis, Treatment, Prevention and Control [2] was followed by the WHO Handbook for Guideline Development [3], which stressed specifically the need for high-level evidence when developing guidelines, particularly through systematic literature reviews. The importance of systematic reviews for linking research and practice was also highlighted by others [4], with one [5] stating “policymakers need systematic reviews that are policy relevant, rigorous, and translatable to their local context, actionable, timely and well communicated”. With this in mind, WHO/TDR together with the WHO/NTD (Department for Neglected Tropical Diseases) and WHO Regional Offices set out to develop an evidence-based handbook [6] for early dengue outbreak detection and response. The project was financially supported by a grant from the European Commission (grant number m281803) to the IDAMS network (www.idams.eu) within the 7th Framework Programme and by TDR/WHO.

Accordingly, this handbook is not intended to be a direct implementation guideline but a framework for developing a national plan, requiring local adaptations to acknowledge fine-scale programme components. The latter point takes into account that contingency response planning requires consideration and incorporation of numerous contextual details such as recognition of the structure of the health and vector control services, available infrastructure and budget, human resources, willingness of staff to cooperate, and many others. Here we present an outline of the handbook, summarizing the main components of a national contingency plan for dengue outbreaks and indicating the key elements that are evidence-based and those that require further research efforts.

## Methods

The development of this evidence-based handbook for dengue contingency planning used a step-wise approach. The first step established an overview by identifying knowledge gaps and commissioning new systematic literature reviews covering the following topic areas: a) dengue vector control [7–16] b) outbreak response [17]; c) dengue disease surveillance [18, 19] and dengue vector surveillance [20]; and d) economic aspects [21].

In a second step, mixed (qualitative and quantitative) research methods were used to identify a) factors leading to the success or failure of national dengue control programmes, b) decision-making that resulted in the declaration of a state of emergency, c) stakeholders`perceptions of their contingency plans, and d) gaps regarding the practical application of contingency plans. These studies were conducted in Bolivia, Brazil, Cambodia, Indonesia and Thailand [22] and were complemented by a comparative analysis of dengue contingency plans from 13 countries [23]. Finally, a multi-country study was conducted that assessed dengue contingency planning and outbreak management in 10 countries [24]. The country selection process varied from study to study based on the dengue burden, information available for the information searched, willingness to participate or a history of recent dengue outbreaks, where appropriate.

In the third step, a retrospective analysis of the predictive ability of variables to warn of forthcoming outbreaks was conducted. Epidemiological and meteorological variables were analysed using datasets from Brazil, Dominican Republic, Malaysia, Mexico and Vietnam [25]. These were selected based on dengue endemicity, dengue burden and those countries with a recent history of dengue outbreaks. In common with the existing scientific literature, the model identified a number of variables that could be used to predict dengue outbreaks with sufficient sensitivity and relatively few false alarms. This model is currently being evaluated in a prospective feasibility and cost-effectiveness study in Brazil, Malaysia and Mexico, as part of an evaluation of a staged response system, designed to gradually implement timely interventions in response to weak or stronger alert signals.

In a last step, we developed a computer-assisted early warning system designed to run on a wide variety of platforms such as Microsoft Excel, STATA, R and SPSS. Such software was developed to build capacity in countries that currently lack the resources to implement predictive dengue technologies. A user-guide was prepared to describe and explain the early warning system, how to use it to identify potential alarm signals at the district level, and how programme managers might use these indicators to provide timely evidence-based alerts to subsequent dengue outbreaks. These developments can equip regional epidemiologists with the technical capacity to rapidly obtain the information required to formulate timely outbreak response.

NB: A formal assessment of quality of evidence of the included literature was not performed in this paper—this article describes the developmental process of the handbook. The material used for the development of the handbook, however, included the highest available evidence for each subsection: a) Guidelines and Handbooks (2,3,26 and 27), b) Systematic Reviews and Meta-analysis (7–22), c) RCTs/cRCTs (28), d) Cohort Studies (29–32), e) Mixed-Method Study Designs (22–24,33 and 34), f) Others (primary research–non controlled and reviews-non systematic) (4,5, 25, 34, 40-67), and g) Reports (1,68–70).

## Results

Successful outbreak detection (the term “outbreak” is used here synonymously with “epidemic”) and response is reliant on a representative and timely surveillance system reflecting the transmission of disease; that is, an effective alert mechanism linking surveillance data to the best possible evidence-based and cost-effective response strategies. The main purposes of a surveillance system are to a) monitor and document disease trends and b) detect outbreaks at an early stage. A contingency plan links these elements together and describes additionally the timing and response actions to be taken when an outbreak is imminent or has begun. In the following sections, we highlight different aspects of contingency planning and provide detailed information on each component.

### Timely contingency planning

In a comparison of existing practices in 10 countries in Asia and Latin America [24], outbreak response plans varied in quality and comprehensiveness, particularly regarding early response measures as well as detailed specifications of actions to be taken. Harrington et al. [23] compared 13 country contingency plans for dengue from Asia, Latin America and Australia, and one international plan by the World Health Organization. The authors found that outbreak governance was weak, in part due to a lack of clarity of the roles of stakeholders, poor surveillance contributed to delays in response, there was a lack of combining routine data with additional alerts, and the absence of triggers to initiate an early response. Frequently, an outbreak was undefined and early response mechanisms based on alert signals were neglected. Therefore it was concluded that a model contingency plan for dengue outbreak prediction, detection and response, including resource planning, training, monitoring and evaluation, could help national disease control authorities to develop their own more detailed and functional context-specific plans. Badurdeen et al. [24] also found that information on dengue was based on compulsory notification and reporting (“passive surveillance”), coupled with laboratory confirmation (in all participating Latin American countries and some Asian countries) or by using a clinical syndromic definition. Seven countries [24] had sentinel sites with active dengue reporting, and some also had virological surveillance. Six countries had a formal definition for dengue outbreaks, distinguishing them from seasonal incident peaks. Countries collected data on a range of warning signs that could identify outbreaks early, but none had developed a systematic approach to identify and respond to the early stages of an outbreak. Through discussions at an expert meeting, suggestions were made for the development of a more standardised approach in the form of a model contingency plan, together with agreed upon outbreak definitions and country-specific risk assessment schemes, in order to initiate timely response activities [24].

### Surveillance systems for outbreak preparedness

#### Surveillance systems and contingency plans

**Fig 1:**
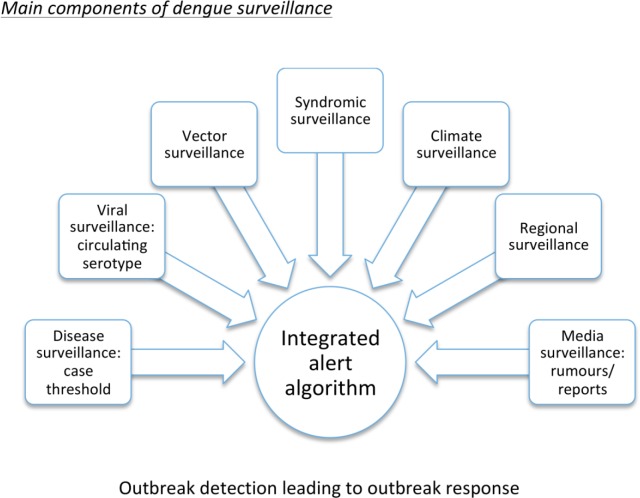
Main components of a dengue surveillance system.

The main components of a dengue surveillance system are summarised in Fig 1. The evidence for their relative value and usefulness is discussed below. Runge-Ranzinger et al. [18, 19] systematically reviewed the usefulness of dengue disease surveillance for outbreak detection and programme planning. Four cohort-based studies [29–32] revealed remarkably high levels of under-reporting in the surveillance systems by calculating “expansion factors” (e.g., how many more cases exist in addition to reported cases). Such high levels of underestimated caseloads hamper the prediction of outbreaks and several studies [35–40] demonstrated that enhancement methods such as laboratory support, sentinel reporting and staff motivation contributed to improvements in dengue reporting, and thus to a more precise, real-time picture of dengue expansion. Alert signals used for syndromic surveillance that are potentially useful in an early warning system are described below under point Four.

In addition to these findings, qualitative research on dengue surveillance and control programs [22, 33, 68] identified several issues that resulted in low sensitivity of case detection, including relying on only a clinical assessment for dengue diagnosis, low patient demand for services, low specificity of the DF/ DHF/ DSS case classification, limited acceptability of the monitoring system at all levels, and case reporting limited to the public sector, to certain age groups, or to in-patient cases. Recommendations from the authors suggest that timeliness in reporting could be improved by: 1) establishing a common understanding on the purpose and objectives of surveillance across all stakeholders; 2) using simplified and standardized case definitions and improving dengue case classifications; 3) improving feedback of reported data to stakeholders; 4) ensuring consistent data flow and clear reporting channels; 5) creating additional active, sentinel and syndromic surveillance based on a clear rationale; 6) using data from virological and serological surveillance; 7) conducting research on appropriate thresholds/ alert indicators or risk assessment tools for dengue outbreak detection, and 8) ensuring that surveillance data, alert mechanisms and evidence-based response are linked and embedded in proper contingency planning.

Information about the circulating serotype/genotype should be documented and used for surveillance purposes. According to Harrington et al. [23], a national contingency plan should state precisely how laboratory surveillance would function during an outbreak. For example, will laboratory surveillance just be used to confirm an outbreak or will it be performed continuously throughout an outbreak? What tests should be used and to whom should the results be sent? Details of laboratory-specific issues to be considered in country dengue contingency plans are: 1) laboratory confirmation of reported cases, 2) how to report positive results directly to the surveillance system, 3) details for viral isolation, PCR, NS-1, ELISA, serological confirmation by IgM and IgG, use of rapid diagnostic tests, storage and transport of samples as appropriate (see WHO [2]), 4) purpose of tests, test results and their interpretation, 5) a flowchart describing the timing of tests and destination of samples, 6) laboratory-specific processes of outbreak investigation and confirmation, 7) quality control, training and capacity building, and 8) prevention of stock-outs.

#### Definition of a dengue case

For early detection of dengue outbreaks, the definition and classification of a dengue case is important. However, clinical diagnosis of a dengue case leading to the diagnosis of “probable dengue” is almost impossible because of a number of similar febrile conditions. The 2009 WHO [2] case classification suggests a case definition that can be used with or without laboratory parameters. It also suggests a distinction between dengue and severe dengue, which is important for clinical management but also for epidemic preparedness. This allows a rough estimate of the clinical services necessary to cope with a large-scale dengue outbreak and facilitate triage processes. Horstick et al. [8] compared the 1997 and 2009 WHO dengue case classifications in a systematic review. The authors found that use of the 2009 WHO dengue case classification resulted in determination of severe dengue with a sensitivity between 59–98% (88–98% within the four prospective studies) and a specificity of 41–99% (99% in the four prospective studies) comparing to the 1997 WHO classification: sensitivity 24.8% - 89.9% (24.8%/74%: prospective studies), specificity: 25%/100% (100%: prospective study). It was concluded that the 2009 WHO classification had clear performance advantages for clinical and epidemiological use when compared with the 1997 classification.

#### Vector surveillance

A systematic review by Bowman et al. [20] investigated the usefulness of entomological indicators as outbreak predictors. Eleven of eighteen studies included in the review generated *Stegomyia* indices from combined larval and pupal data while only three studies reported adult vector data. Of thirteen studies that investigated associations between vector indices and dengue cases, four reported positive correlations, four found no correlation and five reported ambiguous or inconclusive associations. Additionally, six of seven studies that measured Breteau indices reported dengue transmission at levels below the widely accepted threshold of 5. Bowman et al. [20] found there was little evidence of any quantifiable association between vector indices and dengue transmission that could be used reliably for outbreak prediction and that single values of the Breteau or other indices were not reliable universal dengue transmission thresholds. The authors recommended further studies using more appropriate study designs, e.g., standardized sampling protocols that adequately consider dengue spatial heterogeneity, and less reliance on universal thresholds; historic localised vector abundance metrics are considered a more reliable indicator of fluctuation and risk. Additionally, the authors found that operational issues of routine vector surveillance were often hampered by a lack of resources, lack of involvement of local level personnel in decision-making, limitations in supervision, increasing vector resistance to insecticides, and difficulty in the interpretation of entomological indices [24].

### Outbreak definition

Among the systematic reviews performed to date, considerable variation was observed in the number and application of outbreak definitions, and definitions have been numerous, non-standardised and inconsistently applied [24]. In order to ensure that an early warning system for dengue outbreaks is effective, efficient and timely, outbreak definitions must be able to distinguish between true outbreaks and seasonal increases in dengue. Therefore, outbreaks were defined as caseloads of an order much larger than would otherwise be expected during the respective season and/ or occurring in unexpected locations. This task is complex but has been somewhat simplified by the use of the Endemic Channel. Outbreak definitions defined using the Endemic Channel often base thresholds on 2 standard deviations (SD) above the mean number of historic dengue cases, which closely reflects the 1.96 SDs used in confidence estimates to capture 95% of the variation about the mean. However, such values are often applied across large spatial dimensions, resulting in the loss of information that may be reflective of the localised transmission dynamics inherent to dengue [25]. Considering this, models need to be parameterised according to the context [41]. In support of this evidence, Bowman et al. [25] also found that the multiplier of the standard deviation may be context-dependent and reported that 1.25SD could be used as an efficient multiplier. Brady et al. [34] modelled five approaches to define an outbreak using different summary statistics (i.e., recent mean, monthly mean, moving mean, cumulative mean, and fixed incidence threshold). The authors reconfirmed that outbreaks remain highly heterogeneous, in part due to location-specific transmission factors but also due to the methodologies used to define the outbreaks.

In summary, outbreak definitions may need to be spatially stratified, with consideration given to available contextual data and summary statistics, and include operational perspectives to best identify the most important stages of an outbreak in order to ensure a timely response. Until consensus is reached on the most appropriate method to define outbreaks, definitions using simple approaches such as the Endemic Channel should not be discounted. Although outbreak definitions require further empirical work, they remain accessible to both programme managers and regional epidemiologists alike, and if applied at relatively fine scales offer a useful tool for outbreak detection, planning and response [25].

### Alarm signals for outbreaks

Syndromic surveillance [69] may contribute important data on alarm signals in early warning systems for dengue outbreaks. A number of variables that provide predictive warning have been identified and include the rate of school absenteeism [42–44], the volume of internet-based health inquiries [45], the malaria negative rate in fever patients [46, 47], non-specific laboratory requests (as malaria negativity rates or as thrombocytes requested), and fever alerts or use of clinical syndromic definitions [48–51] and the proportion of virologically confirmed cases [52, 53]. Runge-Ranzinger et al. [19] also found six studies [52, 54–58] that showed serotype changes were positively correlated with the number of reported cases or with dengue incidence, with lag times of up to 6 months, indicating that a change in serotype may be a predictor (alarm signal) for dengue outbreaks. Three studies [59–61] found that data on Internet searches and event-based surveillance correlated well with the epidemic curve derived from surveillance data, suggesting that this method may be useful to predict outbreaks. Other approaches such as the use of socioeconomic indicators (presence of water and trash collection services) or environmental parameters (e.g., presence of tire repair shops, rainfall, relative humidity) for risk assessment [62]. Modelling tools [63] also have potential, although at this stage they remain either context-dependent or under evaluation.

In order to develop a dengue outbreak alert model, several potential alarm signals were evaluated retrospectively [25]. A simple approach combining the Shewhart method and Endemic Channel was used to identify alarm signals that could predict dengue outbreaks. Five country datasets were compiled by epidemiological week over the years 2007–2013 and these data were split to form a historic period (2007–2011) and evaluation period (2012–2013). To parameterise the model, associations between alarm signals and outbreaks were analysed using logistic regression during the historic period. Thereafter, these associations were combined with alarm variable data during the evaluation period to predict dengue. Subsequently, model performance was described using sensitivity and positive predictive value (PPV) (the proportion of false alarms). Across Mexico and Dominican Republic, an increase in probable cases predicted outbreaks of hospitalised cases with sensitivities and PPVs of 93%/ 83% and 97%/ 86% respectively. In addition, an increase in mean temperature in Mexico and Brazil predicted outbreaks of hospitalised cases, with sensitivities and PPVs of 79%/ 73% and 81%/ 46% respectively. These results were particularly promising as these variables were broadly predictive of dengue outbreaks across different countries, despite the varied surveillance systems, case definitions and localised variation in transmission potential often associated with dengue [25]. Clearly, routine surveillance can underestimate the true burden of disease, however the prediction of cases was not hindered, as the case definition remained consistent throughout the historic and evaluation periods and the systems were accurately reflecting the burden of disease.

### Managerial capacity

Documented effective outbreak interventions and evidence gaps were analysed in a systematic review by Pilger et al. [17]. Different strategies in the organization of outbreak response were identified, showing that control activities for a dengue outbreak need to be multi-sectoral, multidisciplinary and multilevel; they also require environmental, political, social and medical inputs for coordination so that successful activities of one sector are not weakened by the lack of commitment from another. Risk communication is a fundamental element of managing a public health threat by encouraging positive behavioural change and maintaining public trust [26]. Outbreaks can be highly charged political and social events whereby “outbreak declaration and transparency from expert to audience is surrounded by political and economic overtones” [64]. Therefore it is critical that risk communication plans are prepared prior to an event and that individuals serving as spokespersons are provided with training in public speaking and risk communication in order to proactively manage the outbreak response, along with political or other issues that may arise [26].

The logistics of outbreak response activities are challenging. It is important to assess the additional human resources that will be required, both for clinical management of cases and vector control. This includes redistribution of staff, increased staffing levels and extension of work shifts [24, 70]. Overwork and subsequent demotivation of health staff have been identified as likely problems, often caused by increased demands by politicians and the community [7]. Therefore, staff training and preparation for an outbreak in the inter-epidemic period and supportive supervision during the outbreak can help staff cope with excessive challenges during the outbreak [17]. Investment in human resources must come prior to the outbreak, thus outbreak response planning requires a section documenting the activities to be performed in the inter-epidemic period in preparation for an outbreak, as opposed to preventative control. The contingency plan has also to include the “stopping rules”, i.e., when and how to declare the end of the outbreak, halting the outbreak response and continuing with routine interventions.

### Vector control

Horstick et al. [7] undertook an analysis of vector services with two methods: a systematic literature review and case studies that included stakeholder interviews and completion of questionnaires in Brazil, Guatemala, The Philippines, and Vietnam. In the systematic literature review, staffing levels, capacity building, management and organization, funding, and community engagement were found to be insufficient. The case studies confirmed most of these findings, with stakeholders reporting: 1) lack of personnel (entomologists, social scientists and operational vector control staff); 2) lack of technical expertise at decentralized levels of services; 3) insufficient budgets; 4) inadequate geographical coverage; 5) interventions that rely mostly on insecticides; 6) difficulties engaging communities; 7) little capacity building; and 8) minimal monitoring and evaluation. Stakeholders’ doubts about service effectiveness were widespread, but interventions were assumed to be potentially effective with increased resources. The authors highlighted the need for operational standards; evidence-based selection/ delivery of combinations of interventions; development/ application of monitoring and evaluation tools; and needs-driven capacity building. These recommendations are in line with those from Pilger et al. [17], who reported that combining interventions that involved vector control (elimination of larval habitats with community involvement; appropriate use of insecticides in and around houses) and capacity training of medical personnel, in combination with laboratory support, were crucial for the successful control of outbreaks.

For single vector control interventions, systematic reviews are available on peridomestic space spraying [12], *Bacillus thuringiensis israelensis* (BTI) [9], temephos [16], copepods [13] and larvivorous fish [15]. Horstick and Runge-Ranzinger [65] found that: 1) vector control could be effective, but implementation and coverage remained an issue; 2) single interventions were probably not useful; 3) combinations of interventions had mixed results; 4) careful implementation of vector control measures may be most important; and 5) outbreak interventions were often applied with questionable effectiveness.

A systematic review and meta-analysis found that community-based multiple interventions (such as environmental management or clean up campaigns, refuse collection, the formation of community working groups, social mobilization strategies, water covers, and larviciding) can signficiantly reduce vector densities [14]. Results from a cluster randomised controlled trial in Latin America [28] reported reductions in dengue cases following similar interventions. Bowman et al. [14] also reported that house screens on external doors and windows could be protective against dengue transmission, but that there was insufficient evidence from randomized controlled trials to determine whether or not insecticide space-spraying or fogging could impact dengue transmission. Best practices in vector control remain to be defined for any setting (i.e., which tools or methods the community should employ), as well as what constitutes adequate or sufficient coverage in order to impact the vector population and virus transmission. This includes operational aspects, quality of delivery and best combination of interventions for successful vector control during outbreaks.

Bowman et al. [14] also found no evidence that interventions such as mosquito coils, repellents, bed nets, or mosquito traps could reduce dengue incidence. Finally, indoor residual insecticide spraying and approaches involving the use of genetically modified (GM) mosquitoes or the intracellular symbiont *Wolbachia* [66] have considerable potential for dengue vector control, but have not yet been evaluated sufficiently to draw conclusions about their effectiveness.

### Clinical services

Good clinical case management during an outbreak has been crucial in reducing the case fatality of dengue from 10–20% to less than 1% in many countries over the past two decades [67]. The training of health professionals in diagnosis and management, as well as robust laboratory facilities must be prioritized, as this will effectively dictate case management and influence mortality rates. The best ways to achieve successful training may be through hands-on training during ward rounds and case conferences [17]. The importance of emergency resources and funding for outbreak response including clinical supplies has been highlighted as an important element of preparedness and response planning [2, 24]. Badurdeen et al. [24] found that the surge capacity of hospitals with recent dengue outbreaks varied. Hospital outbreak management plans were present in 9 of 22 participating hospitals in Latin America and 8 of 20 participating hospitals in Asia, also highlighting the need for contingency planning. Further information on triage systems, case management and referrals are available elsewhere [27].

## Discussion

**Fig 2:**
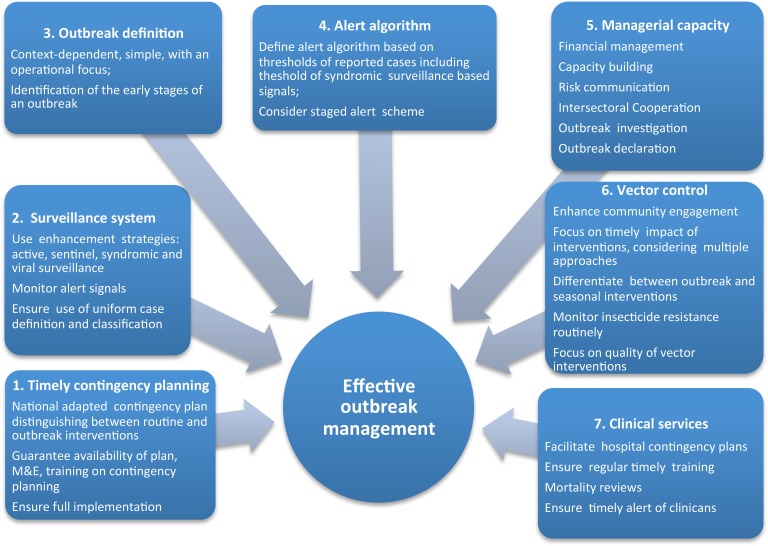
Conceptual framework of dengue contingency planning.

Preparedness planning starts in the inter-epidemic phase and success is dependent on the combination of year-round routine activities, often established in a National Dengue Prevention and Control Plan, up-scaling of routine vector control interventions and communication activities, and timely and systematically initiated additional measures during an outbreak. The proposed handbook suggests seven areas for contingency planning which can either be integrated into the existing national plan or developed as a separate add on. A summary of the recommendations for dengue surveillance, outbreak alert and response are given below in Fig 2.

With respect to timely contingency planning, it is crucial to ensure that a context-specific dengue contingency plan has detailed instructions that allow managers to distinguish between routine interventions required during inter-epidemic periods and those needed during outbreak interventions (i.e., up-scaling of preventive interventions before the start of the “dengue season” vs specific outbreak procedures). The contingency plan should ensure continuity between timely surveillance (including multiple signals), outbreak alerts, and outbreak confirmation based on a clear definition, outbreak declaration, and finally implementation of contingency responses. A key first step is to identify the person/ unit/ agency/ institution responsible for specific activities, to define the roles and responsibilities of each person involved, to ensure the regulatory framework exists to support and facilitate the contingency response, and to ensure that the means and capacity exist to implement the full set of specified contingency activities. This initial planning also takes into consideration the need for human resource preparedness planning for all sectors including distribution of the plan to all stakeholders, instructions for training, and a detailed plan for monitoring and evaluation of preparedness activities and response.

In order to optimize surveillance, a focus on reducing under-reporting and improving reporting timeliness should strengthen routine surveillance systems. It is important to establish a common understanding across all stakeholders on the purpose and objectives of surveillance, to improve feedback of reported data and to provide a clear—ideally electronic—data flow. Enhancement strategies such as sentinel-based reporting, staff motivation, syndromic surveillance, and monitoring additional alarm signals, e.g., virological, serological surveillance, should be included along with use of the simplified and standardized WHO 2009 [2] case classification.

With respect to laboratory support, reporting available laboratory confirmation of cases to the surveillance system is recommended along with information about the circulating serotype/genotype. The laboratory section of the national contingency plan should include details on virus isolation, PCR, NS-1, ELISA, serological confirmation by IgM and IgG, appropriate use of rapid diagnostic tests, storage of samples, and cold chain logistics (see WHO [2]). The purpose of laboratory tests, test results and their interpretation should be described and accompanied by a flowchart that visually depicts the timing of various tests and destinations of samples provided. Laboratory-specific processes of outbreak investigation and confirmation should be defined, including quality control, capacity building, prevention of stock-outs, and the role of different levels of laboratories within the national laboratory network.

The outbreak definition in a national dengue contingency plan should be context-specific and based on the threshold of local historical disease data reported through the national surveillance system. For example, countries may use the Endemic Channel where the threshold is based on z standard deviations (SD) above the mean number of historic dengue cases (currently often z = 2, or according to recent evidence z = 1.25, which is close to the 3rd percentile above the median). Efforts should be made to distinguish between standardized definitions of an outbreak and the local/ national threshold used to initiate outbreak response, considering that large spatial dimensions will result in the loss of information of localised transmission dynamics. In addition to those mentioned herein, additional predictive variables, such as meteorological variables, in particular mean daytime weekly temperature, may be of use in local contexts.

It is crucial to define an alert algorithm based on different alarm signals (epidemiological thresholds plus the use of meteorological data, syndromic surveillance data, laboratory results or perhaps entomological metrics (although there is currently little evidence of quantifiable associations between vector indices)) to increase sensitivity and specificity for predicting forthcoming outbreaks. The outbreak response should be staged in accordance with the identified level of risk (i.e., Initial Response, Early Response, Emergency Response) to ensure that resources are used efficiently and proportionately.

From a managerial aspect, the organization of multidisciplinary response teams, details of logistic/ operational considerations, including standard operating procedures, stopping rules, monitoring and evaluation, staff training prior to an epidemic, resource mobilisation and financial management, legal framework, and recruitment of additional staff during outbreak response, are all important issues for consideration. This includes the training of management personnel in risk communication to ensure timely and appropriate communication within and without the health sector and throughout the broader population. The process of outbreak declaration and risk communication should be well defined and described, so that community engagement and stakeholder involvement contribute to a successful outbreak response at the local level.

With respect to vector control interventions, the focus should be on quality and coverage of vector interventions, as these remain key issues. The involvement of communities in vector control activities, for example “search and eliminate”, increases the likelihood that expanded coverage will be achieved; notably, community-based interventions can impact vector indices, and some evidence exists for an impact on dengue incidence. House screening has demonstrated an impact on dengue incidence and may be an effective intervention against dengue where the context is appropriate. While limited evidence demonstrates a reduction in vector indices following outdoor fogging, there is no evidence yet for an impact on dengue incidence.

With respect to clinical case management, timely alert of clinicians and a hospital outbreak management plan that includes planning for additional beds and staff are essential. Ensuring triage systems for case management, referrals [27] and mortality reviews will improve case management. Disease transmission control in hospitals as well as regular and timely training of hospital personnel must also be considered.

While gaps in knowledge and evidence still remain, much has been accomplished over the past decade that provides a solid basis for evidence-based contingency planning. With the WHO 2009 [2] dengue case classification, improved diagnostic tests and increased national laboratory capacity, stronger national surveillance systems, and ongoing research to develop algorithms that can be used in an operational setting, countries are in a better position to create a dengue contingency plan that reflects their national and local contexts and optimizes available resources for outbreak response.
